# Pathological cell assembly dynamics in a striatal MSN network model

**DOI:** 10.3389/fncom.2024.1410335

**Published:** 2024-06-06

**Authors:** Astrid Correa, Adam Ponzi, Vladimir M. Calderón, Rosanna Migliore

**Affiliations:** ^1^Institute of Biophysics, National Research Council, Palermo, Italy; ^2^Center for Human Nature, Artificial Intelligence, and Neuroscience, Hokkaido University, Sapporo, Japan; ^3^Department of Developmental Neurobiology and Neurophysiology, Neurobiology Institute, National Autonomous University of Mexico, Querétaro, Mexico

**Keywords:** basal ganglia, striatum, calcium image, pathology, network model, cell assembly, Parkinson's disease, Simulation Based Inference

## Abstract

Under normal conditions the principal cells of the striatum, medium spiny neurons (MSNs), show structured cell assembly activity patterns which alternate sequentially over exceedingly long timescales of many minutes. It is important to understand this activity since it is characteristically disrupted in multiple pathologies, such as Parkinson's disease and dyskinesia, and thought to be caused by alterations in the MSN to MSN lateral inhibitory connections and in the strength and distribution of cortical excitation to MSNs. To understand how these long timescales arise we extended a previous network model of MSN cells to include synapses with short-term plasticity, with parameters taken from a recent detailed striatal connectome study. We first confirmed the presence of sequentially switching cell clusters using the non-linear dimensionality reduction technique, Uniform Manifold Approximation and Projection (UMAP). We found that the network could generate non-stationary activity patterns varying extremely slowly on the order of minutes under biologically realistic conditions. Next we used Simulation Based Inference (SBI) to train a deep net to map features of the MSN network generated cell assembly activity to MSN network parameters. We used the trained SBI model to estimate MSN network parameters from *ex-vivo* brain slice calcium imaging data. We found that best fit network parameters were very close to their physiologically observed values. On the other hand network parameters estimated from Parkinsonian, decorticated and dyskinetic *ex-vivo* slice preparations were different. Our work may provide a pipeline for diagnosis of basal ganglia pathology from spiking data as well as for the design pharmacological treatments.

## Introduction

The striatum, the largest part of the basal ganglia (BG), is important in motor control, action selection, and reinforcement learning. It is over 90% composed of medium spiny neurons (MSNs), its only projection neurons. MSNs have been found to be important for multiple cognitive functions including habit learning and formation (Graybiel and Grafton, 2015), multisensory integration (Reig and Silberberg, 2014; Coffey et al., 2016), and motivational control (Haber and Knutson, 2010; Sesack and Grace, 2010; Lobo et al., 2013; Révy et al., 2014; Ikemoto et al., 2015). MSN activity is controlled by feedforward glutamatergic input from the thalamus and cortex, as well as feedforward GABAergic input from striatal interneurons, themselves driven by cortical excitation. Besides this MSNs also receive inhibitory input from the extensive axonal collaterals emmanating from other MSNs (Tunstall et al., 2002; Guzmán et al., 2003; Tepper et al., 2004; Wickens et al., 2007; Wilson, 2007; Taverna et al., 2008; Chuhma et al., 2011; López-Huerta et al., 2013; Moyer et al., 2014; Dobbs et al., 2016; Wei et al., 2017), through which they form a recurrent mutually inhibitory network. The function of this network is not well-understood.

Various pathologies, like Parkinson's disease (PD; Kish et al., 1988), Huntington's disease (Glass et al., 2000), depression (Francis et al., 2015) addiction, and schizophrenia (Simpson et al., 2010) are associated with striatal MSN dysfunction. Parkinson's disease is the second most common neurodegenerative condition in the world (Blesa et al., 2017; Shen et al., 2022). It is caused by loss of over half of the dopamine innervation to the striatum (Burke and O'Malley, 2013). This alters MSN synaptic and cellular properties which is thought to lead to an inbalance of the direct and indirect BG pathways (Day et al., 2006; Kravitz et al., 2010; Gerfen and Surmeier, 2011; Calabresi et al., 2014; Fieblinger et al., 2014; Suarez et al., 2016; Parker et al., 2018) which between them promote and inhibit movement. The inbalance results in motor deficits like tremor, rigidity, hypokinesia and bradykinesia (Lees et al., 2009; Obeso et al., 2017). Dopamine (DA) replacement with L-DOPA provides an effective therapy in the short-term (Foley, 2000; Mercuri and Bernardi, 2005; Lees et al., 2015; De Deurwaerdére et al., 2017), but longer-term it produces a different set of motor problems characterized by abnormal involuntary movements, including chorea and dystonia, termed L-DOPA induced dyskinesia (LID; Ahlskog and Muenter, 2001; Aquino and Fox, 2015; Sharma et al., 2015; Picconi et al., 2017).

**Figure 1:**
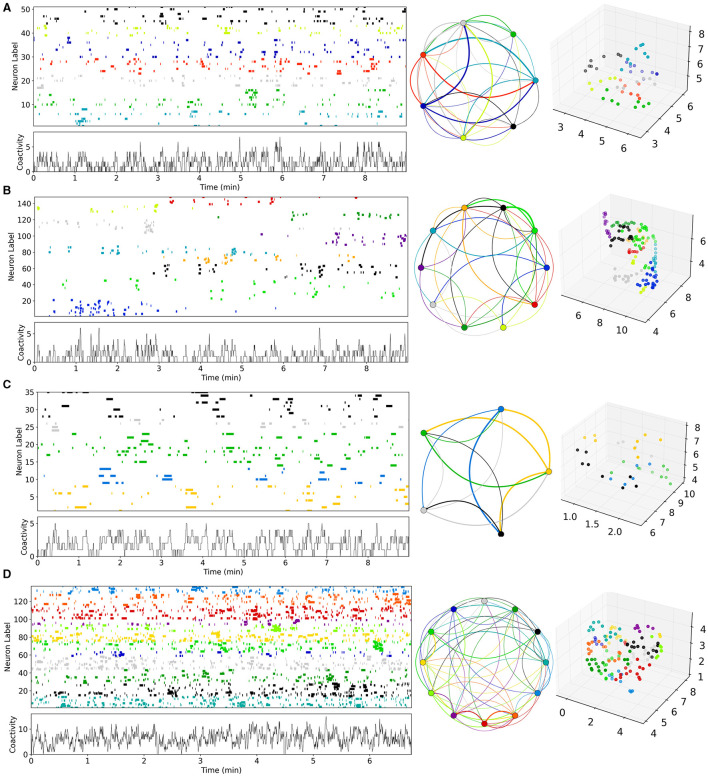
Examples of Ca imaging slice data calculated from data in Pérez-Ortega et al. (2016) and Serrano-Reyes et al. (2022). **(A)** Control (CT), **(B)** decorticated (DEC), **(C)** Parkinsonian (PD), and **(D)** dyskinetic (DYS). Figures have been calculated using code and methods taken from Serrano-Reyes et al. (2022) with identical parameter settings. Time series are clustered using UMAP and the cells colored according to their assigned cluster in the time series (upper left panels) and in the 3D UMAP projections (far right panels). Time series of total activity across cells is also shown (lower left panels). Cluster sequential state transitions are also shown (middle panels) identified using methods and code also taken from Serrano-Reyes et al. (2022). Here the colored circle indicates the cluster, and the lines indicate temporally sequential transitions between active clusters, with line color the same as the initial cluster. Line thickness indicates the quantity of transitions. It should be noted that the appearance of these representations and the quantity of clusters, can depend a lot on the quantity of recorded cells which varies between preparations.

Animal models of PD demonstrate that loss of dopamine is associated with changes to single unit MSN firing patterns (Day et al., 2006; Mallet et al., 2006; Shen et al., 2007) as well as abnormal synchronization and pathological oscillations in cell populations (Raz et al., 1996; Moran et al., 2011; Quiroga-Varela et al., 2013) which accord with symptoms in PD patients (Marreiros et al., 2013; Oswal et al., 2013; Little and Brown, 2014). Sequential neural ensemble activity in striatal cultures (Carrillo-Reid et al., 2008, 2009; Pérez-Ortega et al., 2016; Lara-González et al., 2019, 2022; Serrano-Reyes et al., 2022) and *ex-vivo* brain preparations from different transgenic animal models in multiple brain areas (Ahrens et al., 2013; Lock et al., 2015; Carrillo-Reid et al., 2016; Serrano-Reyes et al., 2020; Pérez-Ortega et al., 2021) has been observed using fluorescent calcium imaging (Figure 1). While control striatal preparations show definite repetitive sequences of neuronal assembly transitions (Carrillo-Reid et al., 2008, 2009), MSN population activity in 6OHDA dopamine depleted striatal preparations, an animal model of PD, seems to become locked into less varied and persistent activity patterns, reminiscent of the pathological hypokinesia and rigidity symptoms shown in PD (Jáidar et al., 2010, 2019; Plata et al., 2013a,b; Pérez-Ortega et al., 2016). On the other hand LID preparations show more frequent and complex cell assembly transitions (Pérez-Ortega et al., 2016; Calderón et al., 2022) than control preparations, which is again reminiscent of the dyskinetic abnormal involuntary movements shown in this condition.

How these alterations in sequential cell assembly activity patterns arise from altered striatal or cortical physiology is not well-understood. In fact a cascade of numerous complex changes occur at the cellular and synaptic levels in PD and LID (Blesa et al., 2017; Shen et al., 2022). Under normal conditions DA modulates intrinsic MSN excitability and synaptic connections, as well as bidirectional corticostriatal synaptic plasticity. It is thought that while DA reduction in PD initially causes an inbalance of activity between BG direct and indirect pathways, subsequently this sets off multiple homeostatic adaptations which try to normalize the inbalance. For example, decreased dopamine levels in models of PD alter dendritic spines on MSNs (McNeill et al., 1988; Ingham et al., 1989; Stephens et al., 2005; Zaja-Milatovic et al., 2005; Day et al., 2006; Villalba et al., 2009; Zhang et al., 2013; Fieblinger et al., 2014; Suárez et al., 2014; Toy et al., 2014; Suarez et al., 2016), which changes cortical and thalamic excitatory transmission. Other studies find that reduced dopamine causes changes in intrinsic cellular excitability and in the density of dopamine receptors (Falardeau et al., 1988; Graham et al., 1990; Decamp et al., 1999; Aubert et al., 2005; Chefer et al., 2008; Sun et al., 2013; Blesa et al., 2017; Shen et al., 2022), produces altered pathological LTP and LTD at cortical-striatal synapses (Centonze et al., 1999, 2001) affecting cortical-striatal excitatory drive, and dramatically alters MSN collateral inhibitory connections (Taverna et al., 2008; Flores-Barrera et al., 2010; López-Huerta et al., 2013; Zhai et al., 2018).

LID is caused by the prolonged dopamine replacement treatment for PD using the DA precursor L-DOPA (Barbeau, 1971; Blesa et al., 2017; Olanow and Stocchi, 2018; Calderón et al., 2022). It was originally aimed at re-balancing the direct and indirect pathways to allow normal processing of cortical and thalamic inputs. But L-DOPA also induces multiple complex persistent changes in the striatal circuit. Excess activity is found in direct pathway MSNs (Huot et al., 2013; Bastide et al., 2015; Yang et al., 2021), and multiple synaptic and cellular pathologies are present such as dysregulated corticostriatal synaptic plasticity (Picconi et al., 2003; Calabresi et al., 2015), altered glutamatergic transmission (Garcia-Montes et al., 2019; Zheng et al., 2020), maladaptive molecular mechanisms in MSNs (Finlay et al., 2014; Eshraghi et al., 2020; Fieblinger, 2021) and altered firing patterns and connectivity (Li et al., 2015; Tamté et al., 2016; Zheng et al., 2020; Yang et al., 2021).

Here we take steps, using computational modeling, to try to understand how aberrant striatal cell assembly population dynamics relates to the complex array of underlying cellular and synaptic pathophysiologies found in PD and LID. In previous work (Ponzi and Wickens, 2008, 2010, 2012, 2013, 2022; Ponzi et al., 2020) we showed that an inhibitory MSN network could generate switching cell assembly activation patterns in good agreement with experimental studies (Carrillo-Reid et al., 2008), providing network parameters, in particular the strength and distribution of excitatory driving and the strength of recurrent inhibition between MSNs, was appropriate for the striatum. However when network parameters are altered away from their true striatal values, various types of pathological cell assembly dynamics can be found (Ponzi and Wickens, 2012, 2022; Ponzi et al., 2020) including patterns resembling those found in 6OHDA and LID slices. Here we quantitatively estimate computational network model parameters from empirical slice data using the deep learning framework, Simulation Based Inference (SBI). Since empirically observed cell assembly patterns (Carrillo-Reid et al., 2008; Jáidar et al., 2010; Pérez-Ortega et al., 2016; Lara-González et al., 2019, 2022) reverberate on exceedingly long timescales of minutes we first extended our previous modeling to include short-term plasticity on the inhibitory collaterals between MSNs, with parameters taken from a recent detailed connectome study (Hjorth et al., 2020), to generate very long dynamical timescales. We varied network parameters and trained the SBI deep net to map features of the dynamical activity patterns generated by the model network, to the network parameters. Subsequently we were able to test our trained deep net on experimental control and pathological slice data to provide insight into physiological changes occurring in PD and LID. We discuss our findings in light of the experimental literature.

## Materials and methods

### Model

**Figure 2:**
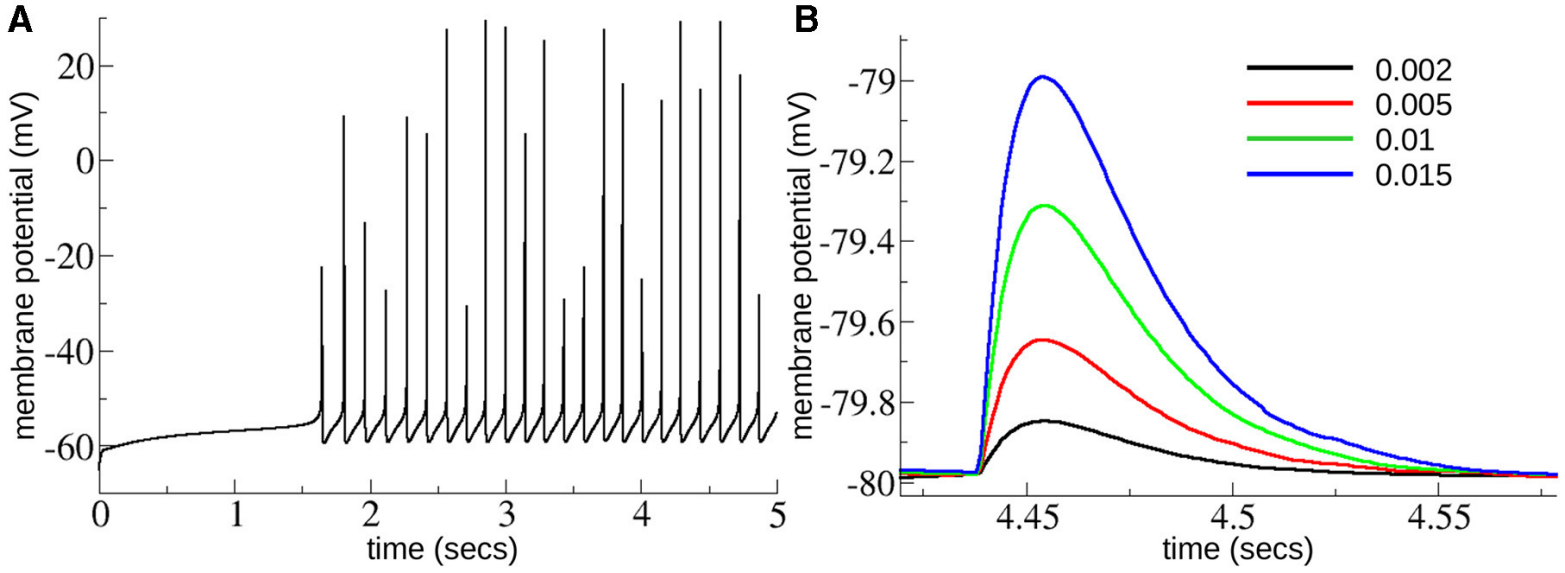
**(A)** Membrane potential trace from MSN cell under excitation level $G_{E}=1.5\times 10^{-5}$, close to the top of the range of *G*_*E*_ explored here. **(B)** Depolarizing IPSPs from an MSN cell held at –80 mV with chloride reversal potential of –40 mV as in Planert et al. (2010) and Hjorth et al. (2020) with several different levels of *G*_*I*_ = 0.002, 0.005, 0.01, 0.015 (see key), across the range of *G*_*I*_ explored here. Experimentally determined mean peak IPSPs (Planert et al., 2010) are between around 0.25 and 0.6 mV and decay over around 40 ms [see Figure 8D(i, ii) in Hjorth et al., 2020].

We made a model of the MSN-MSN network based on a striatal connectome (Hjorth et al., 2020). We only included MSN cells of one type without distinguishing the D1 and D2 subtypes, randomly connected with inhibitory connections. Instead of the detailed cell models used in Hjorth et al. (2020) we used the single compartment cell model used in multiple previous studies (Mahon et al., 2000; Ponzi et al., 2020) including multiple ion-channels, *I*_*Na*_, *I*_*K*_, *I*_*Kir*_, *I*_*Af*_, *I*_*As*_, *I*_*Krp*_, and *I*_*NaP*_, *I*_*NaS*_ which reproduces the delay to first spike property of MSNs, as can be seen in the illustrative membrane potential trace (Figure 2A).

Experimentally the distribution of the number of MSNs connected to any given MSN peaks around 100 but has a long tail (Hjorth et al., 2020). The mean appears to be around 160 (Hjorth et al., 2020). Here synapses between MSNs are established randomly with a a connection probability of 0.4. This connection probability is slightly higher than it should be, which is around 0.3 (Hjorth et al., 2020), but it keeps simulations a bit more tractable since we only need 400 cells to achieve an average of 160 connections per cell which is probably the most important quantity to respect. Thus, all simulations reported here had 400 cells. Simulations lasted for 10 min. The long simulation periods were necessary for comparison with *ex-vivo* Ca slice data recorded over several minutes.

The deterministic version (Fuhrmann et al., 2002) of the Tsodyks-Markram synaptic short-term plasticity model (Tsodyks and Markram, 1997) was used for the MSN-MSN synapses, as in Hjorth et al. (2020),


$$\begin{array}{lll} \frac{dR}{dt} & = & \frac{\left(1-R\left(t\right)\right)}{D}-S\left(t\right)R\left(t\right)\delta \left(t-t_{sp}\right)\\ \frac{dS}{dt} & = & -\frac{S\left(t\right)}{F}+U\left(1-S\left(t\right)\right)\delta \left(t-t_{sp}\right) \end{array}$$

where the short-term depressing variable, *R*(*t*) models the fraction of resources available, and short-term facilitation is modeled by the utilization variable, *S*(*t*), and δ(*t*−*t*_*sp*_) is the presynaptic spike at time *t*_*sp*_. After a spike the synaptic current increases rapidly in proportion to the product *R*(*t*)*S*(*t*) and decays exponentially otherwise. The detailed parameters are as follows: synaptic utilization factor (U), 0.41, depression time constant (D), 222 ms, facilitation time constant (F), 1,859 ms. The synaptic exponential decay timescale was 20 ms, and the GABA reversal potential, -85 mV. These parameters were, as described in Hjorth et al. (2020), taken from Table 1 in Planert et al. (2010). Due to the necessity of very long simulations we were not able to explore multiple different synaptic plasticity parameter values. We took the mean values quoted for U and D, while for F we took the mean value plus one standard deviation. We used the slightly longer F to increase the dynamical timescale within the acceptable range, however a few simulations where F was reduced to the mean value did not seem to be much different. MSN-MSN synaptic delays were uniformly random in the range 1–3 ms, (see Supplemental material for full details).

To estimate the network parameters from slice data we performed 200 network simulations of 10 min each, where the strengths of lateral inhibition between MSNs, *G*_*I*_, and driving excitation to MSNs, *G*_*E*_, were varied. This latter quantity, *G*_*E*_, reflects several factors including the intrinsic MSN cellular excitability, which can be controlled by various modulators such as local dopamine concentration, as well as on the strength of cortical and thalamic excitatory synaptic drive, and also on the strength of feedforward inhibition from striatal fast spiking interneurons. For any given simulation, *G*_*E*_, was uniformly drawn from the range [0, 0.00002] and all MSN cells in the simulation were driven above threshold by somatic current injection (IClamp) with uniform random strength between 0.001305 and 0.001305+*G*_*E*_ which was fixed for the duration of a simulation. For any given simulation, the strength of lateral inhibition, *G*_*I*_, was uniformly drawn from the range [0.002, 0.02] and all MSN synaptic weights were uniformly random in the range 0.3125*G*_*I*_–0.9375*G*_*I*_.

These *G*_*I*_ and *G*_*E*_ ranges cover the acceptable range of IPSP sizes and firing rates we expected to find in the slice data. The maximum firing rate possible occurs when inhibition, *G*_*I*_, is zero and $G_{E}=2\times 10^{-5}$. This generates spiking at around 10 Hz, as shown in Figure 2A. MSNs only spike at higher rates than this when phasically driven in behavior and such rates are not expected to occur in slices. As shown in Figure 2B peak IPSPs at the maximum strength of inhibition, *G*_*I*_ = 0.02, are around 1.5 mV, which is much larger than they are expected to be, while at *G*_*I*_ = 0.002 peak IPSPs are around 0.1 mV which is smaller than they are expected to be. IPSPs are similar to experimentally determined ones (Planert et al., 2010; Hjorth et al., 2020) which peak between around 0.25 and 0.6 mV and decay over around 40 ms, [see Figure 8D(i, ii) in Hjorth et al., 2020]. Model simulation code is available at: https://github.com/adampdp/MSNnetwork. All network simulations are performed using the NetPyNE simulation environment (Dura-Bernal et al., 2019) on the Piz Daint Cray XC40/XC50 supercomputer of the Swiss National Supercomputing Centre (CSCS).

**Figure 3:**
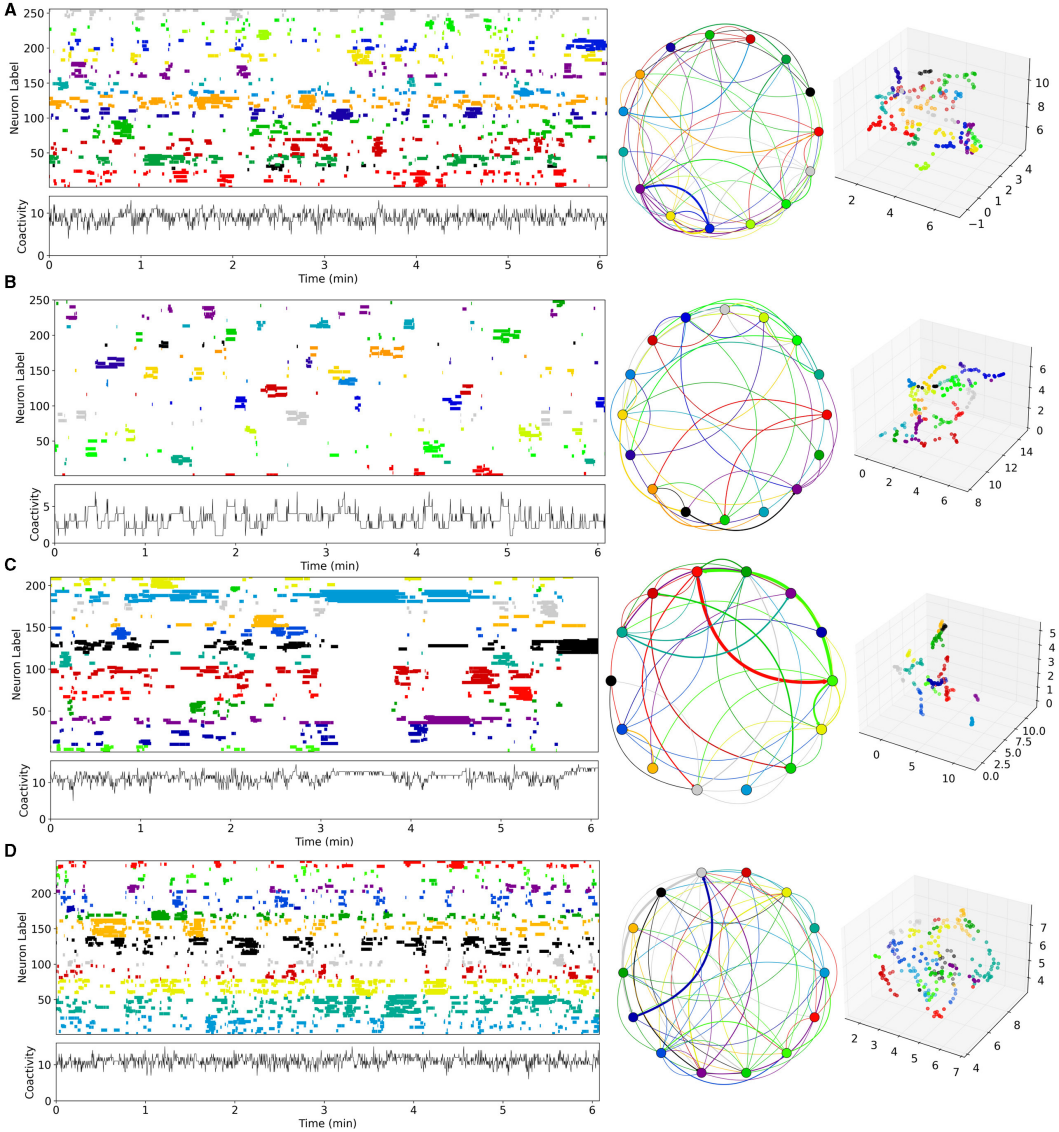
Time series examples from network simulations with different levels of MSN-MSN mutual inhibition, *G*_*I*_, and MSN excitation, *G*_*E*_. **(A)** Best fit control, CT, model, $G_{I}=8.39\times 10^{-3}$, $G_{E}=1.06\times 10^{-5}$. **(B)** Best fit decorticated, DEC, model, $G_{I}=1.96\times 10^{-2}$, $G_{E}=1.02\times 10^{-7}$. **(C)** Best fit Parkinsonian, PD, model, $G_{I}=6.77\times 10^{-3}$, $G_{E}=9.05\times 10^{-6}$. **(D)** Best fit dyskinetic, DYS, model, $G_{I}=8.5\times 10^{-3}$, $G_{E}=1.8\times 10^{-5}$. **(A–D)** Analysis uses UMAP code and methods directly taken from Serrano-Reyes et al. (2022) with identical parameter settings. Figure panels are as described in Figure 1. Model generated 10 min long spike time series are converted to Ca activations with 250 ms time resolution (see Methods). Left panels show the first 6 min for visibility of UMAP clustered cell activations, where the clustering is performed on all active cells (those which fire at least one spike during the recording) over the first 6 min. Middle panels show the corresponding sequential cluster transition graphs. Far right panels shows the corresponding 3D UMAP projections.

To generate the “calcium activity” time series shown in Figure 3, first spike times are recorded from each cell. Next the spike times are turned into spike count time series using 250 ms non-overlapping time bins. Next these spike count time series are binarized according to whether there is at least one spike in each 250 ms window. This provides a method to compare network spiking activity with calcium imaging data which is known to reflect bursts of spiking, with the same 250 ms temporal resolution.

### Simulation Based Inference

We used Simulation Based Inference to estimate network parameters from slice data. From each of the 200 ten minute simulations we extracted summary features. We first removed any cells which did not spike at least once during the 10 minute simulation. Next we converted the cell spikes rasters into binary Ca rate time series with 250 ms bins, as described above. For each of these binary time series we calculated three summary features and used these to train the SBI estimator. The first feature was the mean activity. For each cell we divided the total number of active bins by the total number of bins, then we averaged this quantity across all active cells. Next, for each cell we obtained the sequence of time intervals between consecutive active bins. If a pair of temporally adjacent bins were both active, it was counted as a zero interval. We calculated the average interval and coefficient of variation of the intervals (i.e., the interval standard deviation divided by average interval) for each cell. The second and third summary features were the averages of these quantities across all cells. We experimented with other summary features but found these performed best.

Sometimes simulations fall to fixed-point attractor states, reflecting permanent winners-take-all (WTA) states. An example is shown in Supplementary Figure 1A. Since the full permanent WTA is highly pathological and not expected to be found in experimental slices, we removed simulations which displayed this behavior before training the SBI estimator. To do this we first calculated the total fluctuation of the network activity, Supplementary Figure 1B(i). This is simply the coefficient of variation (CV) of the mean network activity time series. Unlike the three features used to train the SBI estimator, its value strongly depends on the number of active cells. If the network activity falls to a fixed point WTA state this quantity should be zero (after removing a transient period). This occurs more often when excitation, *G*_*E*_, is high and inhibition, *G*_*I*_, is low, Supplementary Figure 1B(i). We removed all network simulations from the SBI training with values <0.09 of this quantity. Notice that the true values for the total fluctuation calculated from the experimental slices, Supplementary Figure 1B(ii), crosses are much higher than found in any simulations, Supplementary Figure 1B(i), and much higher than the values calculated from the best fit network simulations, Supplementary Figure 1B(ii), brown circles. This is because the value of this quantity depends strongly on the number of recorded active cells which is much lower in experimental slices than in network simulations, and also varies strongly across experimental slices. We found it was not feasible to use this quantity as a summary feature for training the SBI estimator because we would need to train the SBI estimator for each experimental slice separately for its specific quantity of recorded active cells (by sampling the appropriate quantity of the cells from the network simulations).

We used Simulation Based Inference code available at: https://sbi-dev.github.io/sbi/. Simulation-based inference offers a powerful avenue for conducting Bayesian analysis without the need for direct numerical evaluation of the likelihood function. Instead, it relies on accessing simulations generated by the underlying model. The core concept of Simulation-based inference (SBI) involves creating a substantial dataset comprising pairs of model parameters and corresponding simulated data. This dataset then serves as training data for artificial neural networks (ANNs), specifically designed to approximate complex probability distributions. Here we used a Gaussian mixture model with parameters comprising means, covariance matrices, and mixture weights for each component. Training involves minimizing the negative log likelihood of the parameterized distribution evaluated at the training data. These ANNs, in turn, enable the approximation of the likelihood, facilitating the generation of posterior samples. SBI allows for the approximation of the posterior distribution of parameters given observed data, using only forward simulations. This circumvents the need to compute potentially intractable log-likelihood functions and their gradients. By incorporating a prior distribution over parameters, a stochastic simulator, and observed data, SBI yields the posterior distribution that best explains the observed data. Following training, these neural networks are then deployed to analyze experimentally observed data, providing an approximation of the posterior distribution.

**Figure 4:**
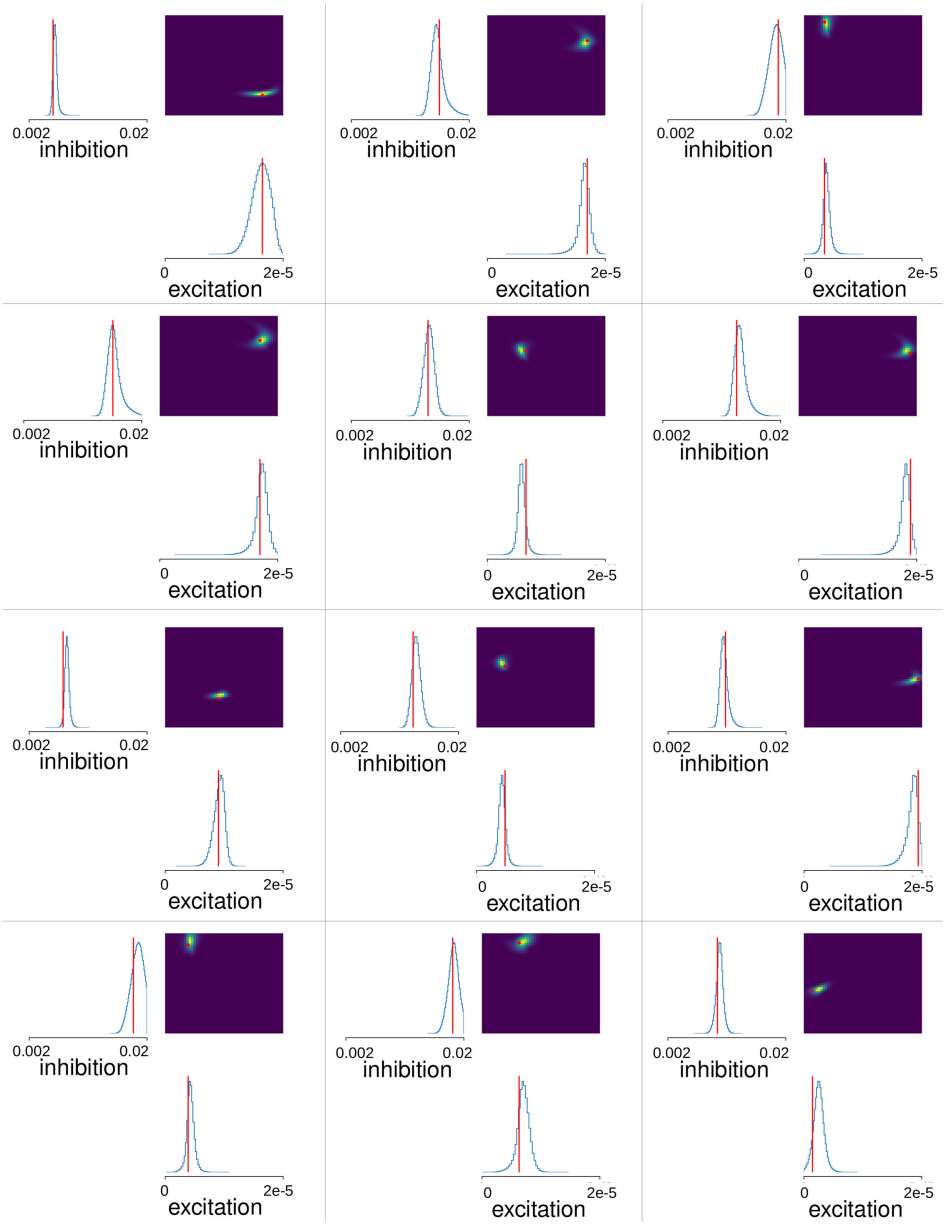
MSN-MSN mutual inhibition, $G_{I}^{*}$, and cortical excitation, $G_{E}^{*}$ levels estimated using SBI for twelve randomly chosen simulations not used in SBI training. The blue lines show the SBI generated marginal posterior distributions for inhibition $G_{I}^{*}$ and excitation, $G_{E}^{*}$. The color plots show the joint posterior distribution. Actual network parameter values, *G*_*I*_, and *G*_*E*_, are shown by the red lines on the marginal distribution plot and the red circle on the joint distribution plot.

**Figure 5:**
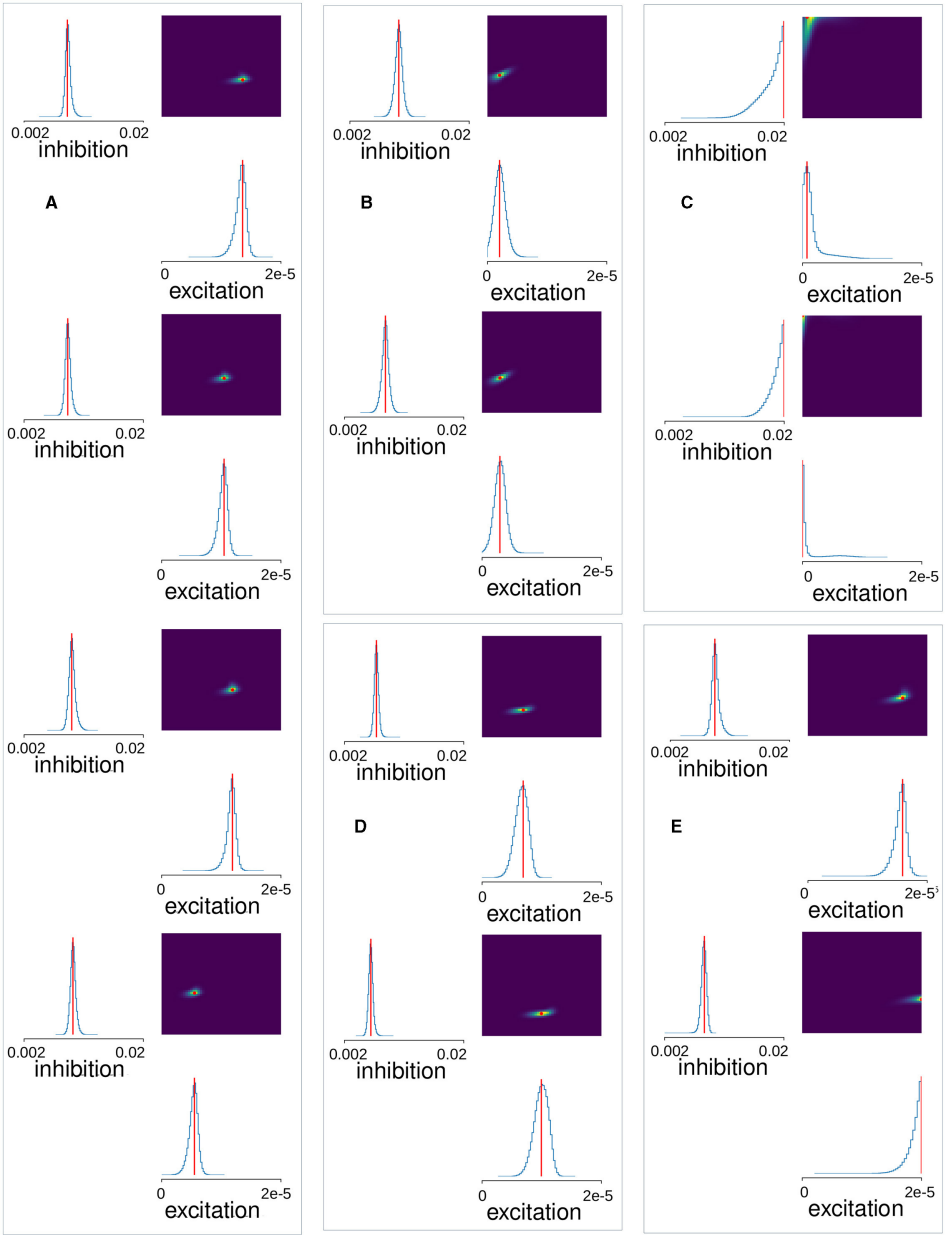
MSN-MSN mutual inhibition, $G_{I}^{*}$, and cortical excitation, $G_{E}^{*}$ levels estimated using SBI for **(A)** four control Ca imaging mouse slice data samples taken from Serrano-Reyes et al. (2022) (CT), **(B)** two examples from another cohort of six control Ca imaging mouse slice data samples (MICE), **(C)** two samples of a decorticated preparation (DEC), **(D)** two samples of a 6OHDA Parkisonian preparation (PD), **(E)** two samples of a dyskinetic preparation after addition of L-DOPA (DYS). **(A–E)** The blue lines show the SBI generated marginal posterior distributions for inhibition $G_{I}^{*}$ and excitation, $G_{E}^{*}$. The color plots show the joint posterior distribution. Red lines and points show the maximum location.

We used Neural Posterior Estimation (NPE) with the three summary features described above for all 151 network simulations with total fluctuation exceeding 0.09. Since results depend on the initial conditions of the training we trained the estimator thirty times on the same set of network simulations. Each time we left out a random selection of twenty simulations as a test set. We calculated the mean-squared error between the true inhibition, *G*_*I*_, and excitation, *G*_*E*_, parameter values for a given simulation and the peak estimated values predicted by the trained SBI estimator, ${\left(G_{I}^{*}-G_{I}\right)}^{2}+900^{2}{\left(G_{E}^{*}-G_{E}\right)}^{2}$, and averaged this over the twenty left-out simulations (the factor 900 is necessary because *G*_*I*_ and *G*_*E*_ vary over different ranges). We selected the winning trained SBI with the minimum mean-squared error from the thirty obtained. Estimated posterior distributions calculated for 12 of the 20 test-simulations for this winner SBI estimator are shown in Figure 4. This trained estimator was applied to the Ca data (Figure 5), using the same summary features to obtain the posterior density over the parameters. One million samples were drawn for the posteriors shown in Figures 4, 5.

To find the best fit network simulation corresponding to each experimental slice condition we calculated the mean-squared error, ${\left(G_{I}^{*}-G_{I}\right)}^{2}+900^{2}{\left(G_{E}^{*}-G_{E}\right)}^{2}$, between the peak estimated parameters for an experimental condition and the true parameters for each network simulation out of the 151 network simulations and used the simulation with the minimum of this quantity for each condition.

### Previously published calcium imaging data

The current work utilizes two data sets: (1) data published by Pérez-Ortega et al. (2016), (2) unpublished data from mice (see below). Here for easy reference we briefly describe the previously published data, for full details see Pérez-Ortega et al. (2016). (CT) Control slices. Horizontal corticostriatal slices (300 μm thick) were obtained from postnatal day (20–36) Wistar rats. Microcircuit activity in the control striatum was activated using 5–8 μM NMDA as the excitatory drive. Without an excitatory drive, the striatal circuit is generally silent in the sense that significant peaks of coactive neurons are virtually absent (Jáidar et al., 2010; Plata et al., 2013a). However, in pathological states, the striatal microcircuit is very active in the absence of any excitatory drive (Jáidar et al., 2010; López-Huerta et al., 2013; Plata et al., 2013b). Pathological conditions examined here were: (DEC) Decorticated striatal microcircuits in which the cortex was detached from the striatum in horizontal slices. In these preparations NMDA was still used as excitatory drive (Pérez-Ortega et al., 2016). (PD) Parkinsonian striatal microcircuits. These preparations were obtained from the striatum of rats lesioned ipsilaterally in the substantia nigra pars compacta with 6OHDA. These preparations were tested for dopamine depletion with behavioral techniques. Rats were tested for evoked turning behavior 8 days after the surgery at postnatal day >23. Animals rotating 450 turns or more were selected for further studies. This behavior corresponds to at least 90% of dopamine depletion. NMDA was not used here as the activity is higher than in the controls in the absence of any excitatory drive (Jáidar et al., 2010; López-Huerta et al., 2013; Plata et al., 2013a,b; Pérez-Ortega et al., 2016). (DYS) L-DOPA induced dyskinetic striatal microcircuits. Animals with severe hemiparkinsonism, which increases the risk of development of LID, were selected. Chronic L-DOPA treatment was administered for 15 days. The development of Abnormal Involuntary Movements (AIMs) was monitored. Rats were considered dyskinetic when they exhibited the main AIMs after chronic application of L-DOPA. These preparations were also recorded in the absence of any excitatory drive (Pérez-Ortega et al., 2016).

### New unpublished calcium imaging data

Additional calcium imaging data were obtained from brain slices from mice (C57BL/6J background) from unpublished experiments using forementioned methodology and preparation according to previous works (Aparicio-Juárez et al., 2019; Calderón et al., 2022) with Calcium Orange (8.2 μM, 0.1% DMSO, 0.67% Pluronic Acid) as calcium indicator. Squared recording sites were in striatum tissue of 750 μm size during spontaneous activity. We detected the rise times of calcium imaging transients, related to burst of action potentials (Carrillo-Reid et al., 2008; García-Vilchis et al., 2019), to obtain the binary spikes raster. For every region of interest (ROIs) we obtained signals, ΔF, corresponding to the average fluorescence of the soma in every frame. Then we applied the following signal processing pipeline (Calderón et al., 2022): (a) estimation of a canonical fluorophore response as a third-degree autoregressive process, (b) noise estimation by wavelet analysis, (c) detrending of fluorescence signals by filtering, and (d) deconvolution by LASSO construction to infer spike activity above 1 standard deviation of the noise: https://github.com/vladscript/finderspiker.

## Results

### Network model displays switching cell assemblies on very long timescales very similar to slice data

Here we aimed to estimate striatal MSN network model parameters from slice data. Some examples of the previously published Ca imaging slice data we used, obtained from Pérez-Ortega et al. (2016) and Serrano-Reyes et al. (2022), are reproduced in Figure 1. These slices are many minute recordings from control (CT), decorticated (DEC), Parkinsonian (PD), and dyskinetic (DYS) preparations. DEC slices have cortical-striatal transmission removed. PD slices come from rats which had previously been 6OHDA lesioned and developed Parkinsonian symptoms. DYS slices come from 6OHDA lesioned rats which were subsequently treated with L-DOPA until they developed Abnormal Involuntary Movements symptomatic of LID. CT and DEC slices are activated by the addition of NMDA, but PD and DYS slices are spontaneously active without this (see Methods and Pérez-Ortega et al., 2016 for details).

As described in Serrano-Reyes et al. (2022) the non-linear clustering algorithm UMAP has been used to visualize the dynamical structure of the Ca imaging data with time resolution 250 ms. Each time series shows all the active cells in the given slice colored according to their cluster assigned by UMAP. The lower left panels show the temporal development of the total activity across all active cells. The UMAP formalism can also be used to project the clusters into three dimensional space. The projection corresponding to each time series is shown in the far right hand panels. Each point is a cell colored according to its assigned cluster. Some distinct clusters in distinct parts of the three dimensional space can be seen. In particular the clusters appear to reactivate repetitively on exceedingly long timescales of many minutes. Sequential reactivation of clusters can be visualized using the circular projections (middle panels). Here each point represents a UMAP cluster while the colored lines between points represent temporal transitions between sequentially activated clusters (see Serrano-Reyes et al., 2022 for details of how the cluster activation levels at any given time point are determined). The lines start at the cluster of their own color and end in a differently colored cluster. The thickness represents how often the particular transition is observed.

While all slices seem to show sequentially activating cell assemblies, the different pathological conditions appear to show quite different types of patterning and cluster structures (Figure 1, Pérez-Ortega et al., 2016; Lara-González et al., 2019; Serrano-Reyes et al., 2022). Activity in the CT slice (Figure 1A), seems to alternate fairly rapidly between repeated cell assembly activations, for example the red and cyan ones. On the other hand, the DEC slice (Figure 1B), seems to show much lower activity levels with longer lived, less repetitive cluster activations. For example the grey, blue, red, dark green and purple clusters each only activate once, each for a couple of minutes. In the PD slice (Figure 1C), activity is weakly repetitive but the quantity of clusters seems somewhat reduced compared to the CT slice (Figure 1A), and the network appears to get locked into a few dominant states for long periods of many minutes, as described in several studies (Jáidar et al., 2010, 2019; Pérez-Ortega et al., 2016; Aparicio-Juárez et al., 2019; Lara-González et al., 2019). For example very slow alternation appears to exist between yellow and green clusters. Activated bursts for individual cells last longer than those in CT slice (Figure 1A), which are much shorter lived. In contrast DYS slices seem to show higher but more “fractured” activity patterns (Figure 1D). Activity is somewhat similar to CT (Figure 1A), but individual cells activate in shorter bursts with less sequential cluster repetitions. Clusters seem to switch more rapidly than CT slices but also more randomly between many different clusters, as previously described (Pérez-Ortega et al., 2016; Lara-González et al., 2019).

To reproduce these dynamics we extended a previously published model of the striatal MSN network (Ponzi and Wickens, 2010, 2013, 2022; Ponzi et al., 2020) to include short-term plasticity between the MSNs. We used a well validated MSN cell model (Ponzi et al., 2020) with a full complement of ion-channels which has been shown to accurately reproduce characteristics of MSN spiking activity, such as the long delay to first spike (Figure 2A). We investigated a 400 cell network and took the synaptic short-term plasticity and connectivity parameters from a recent detailed striatal connectome study (Hjorth et al., 2020; see Methods). We varied the two most important factors which are known to control network model cell assembly dynamics (Ponzi and Wickens, 2010, 2012, 2013, 2022; Ponzi et al., 2020). These are the strength of recurrent inhibition between MSNs, here denoted *G*_*I*_, which controls the postsynaptic IPSP size when a presynaptic MSN spikes, and the MSN excitation level, here denoted *G*_*E*_. Accordingly we varied these two parameters around their physiological values to investigate the dependence of striatal network dynamics on them.

Four example 10 min simulations of model generated calcium activity time series (see Methods), at different levels of *G*_*E*_ and *G*_*I*_, are shown in Figure 3. The cell Ca activity time series are clustered and ordered using the UMAP algorithm, with exactly the same parameters as used for the slice data (Figure 1). As in the slice data various cell cluster activations can be seen. This demonstrates that this MSN network model is capable of generating exceedingly slowly varying dynamics, whereby clusters repetitively switch on timescales of many 10's of seconds. Not all 400 cells are present because cells which do not activate at all during the time period are not shown. The network simulation time series (Figure 3), tend to look denser than the slice time series (Figure 1), because many more cells are shown. The four different examples, at different levels of *G*_*I*_ and *G*_*E*_, show quite different types of dynamical activity, assembly patterns, and clustering (see below).

### Simulation Based Inference accurately predicts network parameter values

Next, we used Simulation Based Inference (SBI) with sequential neural posterior estimation (SNPE; see https://sbi-dev.github.io/sbi/) to fit the MSN network parameters to Ca slice data. A mixture of Gaussians density estimator was trained to map summary features calculated from MSN network generated time series data to the network parameters (see Methods). We performed 200 network simulations of 10 min each, where the strengths of lateral inhibition, *G*_*I*_, and excitation, *G*_*E*_, were varied. Some simulations were rejected due to highly pathological behavior (see Methods). We trained the SBI density estimator on the remaining MSN network simulations after leaving out a further 20 simulations as a test set. Figure 4 shows the SBI parameter estimation for twelve simulations randomly chosen from this test set. The blue lines show the marginal posterior distributions for lateral inhibition, $G_{I}^{*}$, and cortical excitation, $G_{E}^{*}$, (here and in the following ^*^ denotes estimated value, as opposed to actual simulation parameter value) the color plots show the joint posterior density, and the red lines and points indicate the true parameter values, *G*_*I*_ and *G*_*E*_, for the given network simulation. In most cases the joint posterior density is quite sharply peaked around the true parameter values, and close to zero elsewhere, despite the wide range of different parameter settings shown, including simulations close to the borders of the prior parameter ranges. This suggests the SBI parameter estimation procedure is highly effective.

### Estimated IPSP sizes from control slices are close to physiological values

**Figure 6:**
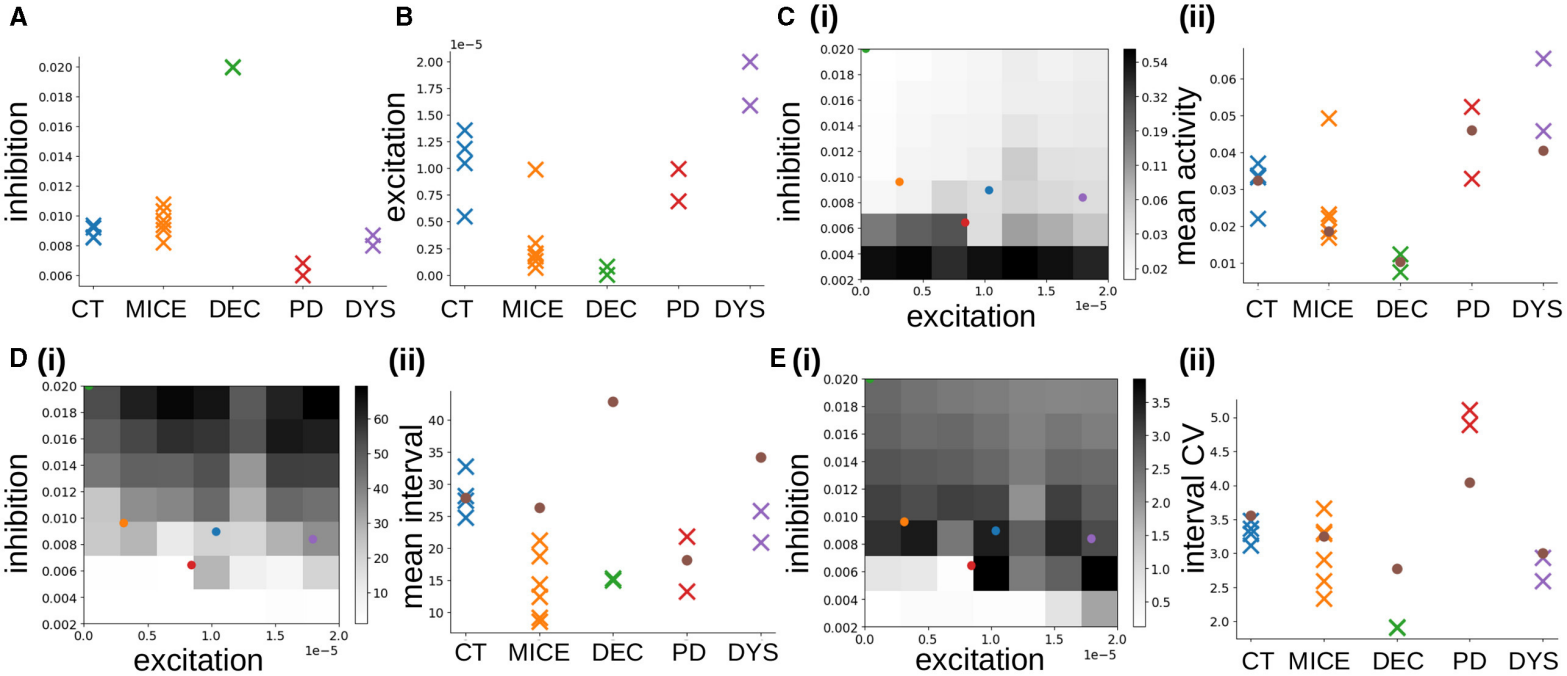
**(A, B)** MSN network parameters estimated from Ca data using SBI for CT, MICE, DEC, PD, and DYS slices. **(A)** Inhibition, $G_{I}^{*}$. **(B)** Excitation $G_{E}^{*}$. **(C–E)** Activity features used in SBI density estimation procedure. **(C)** Mean activity (log scale). **(D)** Mean interval between activations. **(E)** Coefficient of variation of intervals between activations. (i) Dependence of activity features on network simulation parameters inhibition *G*_*I*_ and excitation *G*_*E*_. Colored points indicate the average estimated parameters $\left(G_{E}^{*},G_{I}^{*}\right)$, for the different experimental conditions, blue: CT, orange: MICE, green: DEC, red: PD, purple: DYS (DEC only half visible in top-left corner). (ii) Colored crosses: true feature values calculated directly from the individual experimental slice preparations. Brown circles: feature values calculated from the corresponding best fit simulations shown in Figure 3.

Finally, we calculated the same summary features from the Ca slice data and used the trained SBI density estimator to map these features to corresponding MSN network parameters. Figure 5 illustrates the results of applying this procedure to the Ca slice data. Here the blue lines indicate the marginal posterior density estimates and the red lines and points indicate the maxima of the posterior estimates. The posterior densities for the four control slices (Figure 5A), are all very sharply peaked. Remarkably, they all indicate very similar tightly clustered levels of estimated inhibition $G_{I}^{*}$, of around 0.008. These maximal posterior estimate values are shown in Figure 6A, CT. Interestingly the estimated inhibition level $G_{I}^{*}$, is very close to its physiologically correct value. Indeed, as shown in Figure 2B, peak IPSP sizes around 0.45 mV, the physiologically observed value (Planert et al., 2010; Hjorth et al., 2020), occur between about *G*_*I*_ = 0.006 and *G*_*I*_ = 0.01.

The observation that best fit network models had IPSPs of about the physiologically correct size is highly non-trivial because the SBI density estimator was trained on a wide range of inhibition levels from *G*_*I*_ = 0.002 to *G*_*I*_ = 0.02, which generate a large spread in IPSP sizes (Figure 2B). To investigate this further, we used SBI to estimate the MSN network model parameters from unpublished Ca slices from a different cohort of multiple mice, under the same NMDA activated control conditions. The estimated posterior densities from two of these six slices are shown in Figure 5B, “MICE.” Again the densities are sharply peaked. Figure 6A, MICE shows maximal posterior estimated inhibition $G_{I}^{*}$, levels in this extra group of six Ca slices. Estimated $G_{I}^{*}$ levels are again fairly tightly clustered between about $G_{I}^{*}=0.007$ and $G_{I}^{*}=0.011$ corresponding to peak IPSP sizes of around 0.4 and 0.7 mV which are again close to, but slightly larger than, physiologically observed values (Planert et al., 2010; Hjorth et al., 2020).

Excitation levels for the control groups (Figure 6B, CT, MICE), are more widely spread. The CT group includes three quite closely clustered around $G_{E}^{*}=1.25\times 10^{-5}$, while one has lower excitation. The MICE group includes five clustered with very low excitation, around $G_{E}^{*}=1.5\times 10^{-6}$, but one with higher excitation. Unlike inhibition, it is not possible to compare these estimated values with empirical physiological cortical-striatal (or thalamic-striatal) EPSP sizes because we are here investigating a slice preparation. All slices are activated by the bath addition of NMDA, since the striatum is purely inhibitory and shows almost no activity without it. Therefore we did not include cortical-striatal synapses or spiking cortical input to the striatum network model. MSN cells are excited purely by somatic current injection (see Methods) modeling the bath addition of NMDA. Moreover, MSNs can change their intrinsic excitability levels depending on various modulatory and pathological factors so that they can spike with different rates for any given level of cortical (or thalamic) excitation. Our parameter *G*_*E*_ also reflects these effects. However, since the mean activity is one of the features used to estimate network parameters (see below), providing that the estimated level of inhibition is roughly correct, as we in fact found it to be, the excitation level should also be reasonable since between them these two factors determine the mean spiking rate. Differences in NMDA application could also cause large differences in excitation levels across the two groups, CT and MICE, and within groups. It is to be expected that excitation will be more variable across experiments than inhibition since the inhibition level is a structural characteristic of the striatum while the excitation level can strongly depend on the experimental conditions. During behavior in live animals, levels of excitation arising from cortical and thalamic driving would also be expected to vary strongly, while the strength of lateral inhibition between MSNs is thought to be more determined by fixed synaptic properties and should be less variable.

### Pathological slices clustered in different parameter regions

To see if our findings in control slices are not simply a chance result of the SBI estimation procedure, we also estimated network parameters from the pathological slice preparations. Posterior distributions generated by the trained SBI density estimator for two DEC slices, two PD slices, and two DYS slices, obtained from previously published work (Pérez-Ortega et al., 2016; Serrano-Reyes et al., 2022), are also shown in Figure 5. Densities are again quite sharply peaked, except for the decorticated preparations (Figure 5C). Interestingly posterior densities tend to be quite similar within each group, but can differ quite strongly between groups. Remarkably, like the control slices (Figure 6A, “CT,” “MICE”), we find that maximal posterior inhibition values $G_{I}^{*}$ (Figure 6A, “DEC,” “PD,” and “DYS”), are quite strongly clustered within each group, but differ between most groups. Peak excitation values, $G_{E}^{*}$ (Figure 6B), are more widely spread but clustering within groups, with different levels of excitation between groups, is still evident.

DEC preparations (Figure 5C), show very high inhibition, $G_{I}^{*}$, and very low excitation, $G_{E}^{*}$, as confirmed by peak values for these quantities [Figures 6A, B, “DEC”; the inhibition values, $G_{I}^{*}$, for the two samples (Figure 6A, “DEC”), are indistinguishable in the figure]. Although colocalized across samples, their estimated densities are much broader than the other preparations (Figure 5C), and they are also close to the borders of the parameter priors we used to train the SBI, suggesting lower confidence in the estimated peak values.

Estimated densities from PD preparations (Figure 5D), are as sharply peaked as control ones (Figure 5A), with similar peak excitation values, $G_{E}^{*}$ (Figure 6B, “PD”), but peak inhibition values, $G_{I}^{*}$ (Figure 6A, “PD”), are lower. In fact estimated peak inhibition values for both PD slice preparations (Figure 6A, “PD”), are lower than all ten estimated peak inhibition values for control slices, CT and MICE (Figure 6A, “CT,” “MICE”), as well as all four DEC and DYS slices (Figure 6A, “DEC,” “DYS”), suggesting that reduced inhibition in 6OHDA slices is a significant finding.

Estimated densities from DYS preparations (Figure 5E), are also quite sharply peaked, although in one sample estimated excitation is on the border of the prior parameter range used to the train the SBI density estimator. Peak inhibition values (Figure 6A, “DYS”), are clustered, but in contrast to the PD slices, are similar to those from control animals (Figure 6A, “CT,” “MICE”). On the other hand, both DYS peak estimated excitation values (Figure 6B, “DYS”) are higher than all of the CT, MICE, PD, and DEC slices (Figure 6B, “CT,” “MICE,” “PD,” and “DEC”), suggesting their excess excitation is also a significant finding.

### Predicted network simulation dynamics resembles experimental slices

Given that estimated parameter values were quite strongly clustered within groups, but varied between groups (Figures 6A, B), we wondered what network dynamics these parameter values predict for each of the groups. For each group we calculated the average of the maximal estimated inhibition, $G_{I}^{*}$ and maximal estimated excitation, $G_{E}^{*}$, levels across all the slices in the group, and searched our 200 network simulations for the simulation with most closely matching parameter values for each group using the mean-square distance (see Methods). The “winner” simulations for CT, DEC, PD, and DYS preparations are the ones shown in Figure 3.

The cluster activation patterns shown by these network simulations admit many similarities with the corresponding experimental slice data (Figure 1). Compared to the best fit CT simulation (Figure 3A, left panel), the best fit DEC simulation (Figure 3B, left panel), shows much sparser activity. Cell clusters are still seen but they activate much more rarely with large silent periods, and without strongly apparent sequential patterns. Some cell clusters, such as the dark blue colored one around cell index number 150 (Figure 3B, left panel), activate strongly only once during the 6 min period shown. Cluster transition graphs (Figure 3B, middle panel), appear to show fewer strong “loops” than the best fit CT simulation (Figure 3A, middle panel). For example the best fit CT simulation (Figure 3A, middle panel), shows a fairly strong recurrent “circuit” composed by the dark blue cluster and purple cluster. These observations are to a certain degree evident in the experimental data (Figures 1A, B). Several fairly strong recurrent loops can be seen in the CT experimental slice (Figure 1A, middle panel), but not in the DEC preparation (Figure 1A, middle panel), while the DEC activity is much sparser than CT activity with large silent gaps between cluster activations, as described above.

Compared to the best fit CT simulation (Figure 3A, left panel), the best fit PD simulation (Figure 3C, left panel), shows larger, longer-lived persistent, and “locked-in” clusters. When such clusters are active the rest of the network is mostly silent. The transition graph is dominated by a strong recurrent loop (Figure 3C, middle panel, red, and green). These findings are recapitulated in the experimental slices. The PD experimental transition graph (Figure 1C, middle panel), is also dominated by a strong recurrent loop between yellow and green clusters, and the PD clusters (Figure 1C, left panel), are much longer lived than the CT experimental slice (Figure 1A, left panel). Finally the best fit DYS simulation displays higher activity levels with many shorter fractured bursts (Figure 3D, left panel), and many weak cluster transitions (Figure 3D, middle panel), compared to the best fit CT simulation (Figure 3A), and again these features are also found in the experimental DYS slice (Figure 1D).

### Control slices are close to a network activity transition

The resemblance of sequential cluster structure of the best fit network simulations to the corresponding experimental slices is a highly non-trivial finding because we did not use any clustering related features to train the SBI density estimator. In fact, because the number of recorded cells varies strongly across experimental slice preparations, we were only able to use features derived from single cell activity, rather than cross-cell correlation based features (see Methods). The features we used were: the mean activity, the mean interval between subsequent activations, and the coefficient of variation (CV) of the intervals between subsequent activations. These quantites were averaged across all active cells to provide the three features for SBI parameter estimation. If the individual cell activation time series in a given network simulation, or in a given experimental slice, are temporally shifted with respect to each other, these feature values remain unchanged. This demonstrates that the sequential cluster structure in the population can be revealed by single cell activity characteristics in this network model.

The dependence of these three features on network model inhibition, *G*_*I*_ and excitation, *G*_*E*_, parameters, is shown in Figures 6C–E(i). The mean activity [Figure 6C(i)], decreases with increasing inhibition *G*_*I*_, and at higher inhibition also with decreasing excitation *G*_*E*_, albeit not as strongly. The mean interval between activations [Figure 6D(i)], increases with increasing inhibition and more weakly with increasing excitation. The behavior of the interval CV [Figure 6E(i)], is non-monotonic (Ponzi and Wickens, 2013, 2022; Ponzi et al., 2020). It increases as inhibition is decreased from high values, before a transition around *G*_*I*_ = 0.006 occurs and it decreases suddenly to very low values, or zero. In fact the transition occurs when the network changes from a strongly fluctuating state with high interval CV, to a WTA fixed point state with low or zero interval CV. In the WTA state the network activity is dominated by a set of regularly firing cells which permanently suppress all the others into silence. An example of a such a “pathological” simulation in the WTA regime is shown in Supplementary Figure 1A.

We found the three features [Figures 6C–E(i)], represent the inhibition, *G*_*I*_ and excitation, *G*_*E*_, space in different ways, and are sufficient to train the SBI density estimator to accurately map features to parameters (Figure 4). The colored circles in Figures 6C–E(i) show the estimated inhibition $G_{I}^{*}$ and estimated excitation $G_{E}^{*}$, for the different experimental slices, averaged across the slices in each condition. Most interestingly we find that the two control conditions, CT, and MICE, and the dyskinetic condition, DYS [Figures 6C–E(i)], blue, orange, and purple circles) all reside in the regime with high interval CV just above the transition to the WTA state [Figure 6E(i)], blue, orange, and purple circles). The strong fluctuations found in this regime are generated by the switching coherent cell assembly dynamics seen in the best fit network simulations for CT and DYS conditions (Figures 3A, D). In previous work (Ponzi and Wickens, 2012, 2013, 2022; Ponzi et al., 2020) we have shown that this regime generates complex dynamics which is optimal for neuronal computation (see Discussion). On the other hand the decorticated condition, DEC [Figures 6C–E(i), green circle] resides on the border of the *G*_*I*_, *G*_*E*_, parameter space, at very low excitation and very high inhibition and, as described above, its true parameter values may lie outside the prior parameter distribution. Its very low excitation and very high inhibition are responsible for the short lived bursts and very long silent periods found in its corresponding best fit network simulation for the DEC condition (Figure 3). Intriguingly, the reduction in estimated inhibition $G_{I}^{*}$, we found in the PD experimental slices [Figures 6C–E(i), red circle] situates the PD experimental slices much closer to the border of the pathological WTA regime [Figure 6E(i), red circle]. It is this proximity to the WTA regime which produces the metastable cell assemblies which silence the rest of the network for extended periods, as the system transiently visits the vicinity of WTA states.

Figures 6C–E(ii), colored crosses show the true values of the three features calculated directly from the experimental slices. The brown circles [Figures 6C–E(ii), brown circles], show the feature values calculated directly from the best-fit network simulations in each condition, shown in Figure 3. True features values for the two control conditions, CT and MICE [Figures 6C–E(ii), blue, orange] are very close to the feature values indicated by their corresponding estimated excitation, $G_{E}^{*}$, and inhibition $G_{I}^{*}$, levels [Figures 6C–E(i), blue, orange circles], and to the values directly calculated from the best-fit simulations [Figures 6C–E(ii), brown circles]. This confirms that our model is able to accurately reproduce control slice data.

True feature values calculated from the experimental slices in pathological conditions [Figures 6C–E(ii), green, red, and purple], however, can sometimes stray a little from the values indicated by their corresponding estimated excitation $G_{E}^{*}$, and inhibition $G_{I}^{*}$, levels [Figures 6C–E(i), green, red, and purple circles] and from their best fit simulation values [Figures 6C–E(ii), brown circles]. True experimental mean activity levels [Figure 6C(ii), colored crosses] are always in good correspondence with their corresponding best fit simulation values Figure 6C(ii), brown circles]. But in the DEC condition, the true mean interval [Figure 6D(ii), green], is much shorter than the corresponding best-fit simulation mean interval [Figure 6D(ii), brown circle]. The true interval CV for DEC [Figure 6E(ii), green] is also lower than the corresponding best-fit interval CV [Figure 6E(ii), brown circle]. For the DYS condition, the true mean interval[Figure 6D(ii), purple], is a bit lower than its corresponding best-fit estimated value [Figure 6D(ii), brown circle]. On the other hand in the PD condition the true interval CV [Figure 6E(ii), red], is actually higher than its corresponding best-fit estimated value [Figure 6E(ii), brown circle]. Indeed the interval CV in the experimental PD condition acquires a very high value, around five, which is both much higher than the other experimental conditions, and higher than found in any of our network simulations [Figure 6E(i)]. And indeed the PD interval CV estimated by SBI [Figure 6E(ii), brown circle], is close to the maximum value, around four, which can be found amongst the available network simulations (see Discussion).

## Discussion

The findings presented in this paper shed light on the intricate dynamics of the MSN network in the striatum and its modulation under various conditions. Through the utilization of advanced computational techniques, we aimed to elucidate how excitatory and inhibitory neurotransmission governs dynamics generated by this network. We employed a well-validated MSN cell model (Mahon et al., 2000), incorporating a comprehensive array of ion channels, to simulate spike time series. Leveraging the UMAP algorithm, we compared the spiking activity of our model with experimental data, demonstrating the model's ability to faithfully recapitulate the behaviors observed in the slice data. This highlights the utility of our MSN network model as a valuable tool for investigating striatal network dynamics.

Our previous striatal MSN network model (Ponzi and Wickens, 2008, 2010, 2012, 2013, 2022; Ponzi et al., 2020) was extended to include short-term plasticity on the lateral connections between MSNs. The network structure and parameters were taken from a recent connectome investigation (Hjorth et al., 2020) which represents the most thorough computational depiction of the MSN network ever compiled. To keep simulations tractable we used 400 cells which is sufficient to accurately represent a local striatal network (see Methods). We first showed that this detailed network model was capable of generated slowly varying rate fluctuations on exceedingly long timescales of several minutes, extending our previous findings (Ponzi and Wickens, 2010). Slow rate fluctuations were composed by switching population cell-assembly dynamics.

We employed Simulation Based Inference (SBI) with sequential neural posterior estimation to fit MSN network parameters to calcium imaging slice data. Through simulations varying levels of lateral inhibition and cellular excitation, the model was trained to map summary features from slice data to corresponding MSN network parameters. The resulting posterior distributions Figure 4, exhibited sharp peaks, underscoring the precision of the estimation process. However, decorticated preparations displayed broader estimated densities on the boundaries of our parameter ranges, suggesting reduced confidence in parameter estimates for this condition.

The comparison of peak estimated parameter values across different experimental preparations revealed intriguing insights into the excitatory and inhibitory transmission in MSN networks under the different conditions. We were able to find network simulations which were extremely good fits to the control experimental slice data [Figures 6C–E(ii), blue crosses]. Interestingly we found that the peak IPSP size which provided the best fit to control slice data was very close to the physiologically known value of around 0.45 mV (Planert et al., 2010; Hjorth et al., 2020). We were also able to find network simulations which provided reasonable fits to the pathological slice data. We found that IPSP sizes were well-grouped in distinct clusters for each of the three manipulations in the slices from pathological animals. PD slices showed substantially smaller IPSP sizes than control, while decorticted slices had larger IPSP sizes. Excitation levels had slightly larger spreads than inhibition across all groups, while dyskinetic preparations showed substantially higher excitation and decorticated preparations relatively low excitation.

Due to computational constraints we were only able to vary two parameters, IPSP amplitude and excitation level. In reality IPSP sizes depend on several factors such as presynaptic GABA release and postsynaptic uptake. They also depend on the quantity or location of synapses made by a presynaptic cell on a postsynaptic one. However a change in IPSP size is not equivalent to a change in connection probability. Here, on average, and MSN is presynaptic to about 160 other MSNs. If the IPSP size were halved, all 160 would receive an smaller IPSP when the presynaptic MSN spiked. On the other hand if connection probability is halved, 80 would receive a full sized IPSP and the other 80 nothing. These can have quite different effects at the level of network dynamics. In future studies it will also be important to vary connection probability. We also varied the excitation strength. An increase in this quantity means that the MSN cell would spike at a higher rate for any given level of MSN lateral inhibition. This could reflect an increase in the spike rate from cortical or thalmic excitatory neurons. It could also reflect a decrease in feedforward inhibition from striatal interneurons. Alternatively it could reflect a change in excitability of the cell, for example caused by activation of D1 and D2 receptors when dopamine levels change. Any increase in excitation we found could reflect an upregulation of D1 receptors or down regulation of D2 receptors, for example.

Our model is of course simpler than the real striatal MSN network. In particular we do not include D1 and D2 cell types whose differential dysfunction is known to be highly relevant in both Parkinsonian 6OHDA lesioned animals and LID animals (Blesa et al., 2017; Shen et al., 2022). Unfortunately since the slice data does not include D1 versus D2 labeling, even if the model included these two cell types, we would not be able to use features derived from their differential activations to estimate network parameters. Under normal conditions DA modulates intrinsic MSN excitatability and synaptic connections between MSNs, as well as bidirectional corticostriatal synaptic plasticity. It is thought that DA reduction in PD at first causes an inbalance between the BG direct and indirect pathways which then sets of a cascade of multiple complex homeostatic adaptations to help normalize this inbalance. For example decreased dopamine levels in models of PD alter dendritic spines on MSNs (McNeill et al., 1988; Ingham et al., 1989; Stephens et al., 2005; Zaja-Milatovic et al., 2005; Day et al., 2006; Villalba et al., 2009; Zhang et al., 2013; Fieblinger et al., 2014; Suárez et al., 2014; Toy et al., 2014; Suarez et al., 2016). D2 MSNs become hyper-excitable without dopamine since D2R activation has a suppressing effect. In response they reduce cortical and thalamic synapses to reduce excitatory transmission, and decrease intrinsic excitability. Increases in the density of D2 receptors by dopamine depletion is also reported (Falardeau et al., 1988; Graham et al., 1990; Decamp et al., 1999; Aubert et al., 2005; Chefer et al., 2008; Sun et al., 2013). On the other hand, D1 MSNs become hypo-excitable without dopamine and in response up-regulate their somatic excitability. D1 and D2 receptors control LTP and LTD in the presence of DA, and DA absence strongly alters these mechanisms to affect cortical-striatal excitatory drive (Centonze et al., 1999, 2001).

Despite this model limitation several of our findings are in striking agreement with experimental observations. We found a reduction in IPSP size in the PD rats. Studies have found reduced MSN-MSN connection strength in PD (Taverna et al., 2008; Flores-Barrera et al., 2010; López-Huerta et al., 2013; Zhai et al., 2018). Taverna et al. (2008) found marked reductions in connection probability between MSNs, IPSP peak size and area, and strong increases in failure rate, within and between both D1 and D2 cells in 6OHDA lesioned rats. They suggested the reduced IPSP size was largely attributable to reduced GABA release. Short-term plasticity is also known to be affected in 6OHDA mice (Barroso-Flores et al., 2015; Wei et al., 2017) which would also produce changes in IPSP size.

Although we found a reduction in network inhibition, we did not find much change in activity levels in PD compared to control slices (Figure 6C). Indeed activity rate changes occur in opposite ways in D1 and D2 cell types. The most extensive study to date (Parker et al., 2018) found that 1 day after 6OHDA lesion activity rates during rest were increased in D2 MSNs and decreased in D1 MSNs by approximately the same amount. Interestingly 14 days after lesion resting state activity levels were still decreased and increased in D1 and D2 MSNs respectively, in almost exactly equal amounts, but the difference from pre-lesion baseline had decreased somewhat. Thus in good agreement with our findings on average across both cell types activity was not changed from baseline either 1 or 14 days post-lesion during rest. On the other hand, some differences were found during animal motion. However since motion is likely to be associated with dynamic changes in cortical excitation, the slices investigated here are more likely to be representative of the resting state activity. The same study (Parker et al., 2018) also investigated D1 and D2 activity in 6OHDA rats after a large dose of L-DOPA to induce dyskinesia. In this case they found that during rest D2 MSN activity was suppressed compared to pre-lesion baseline while D1 activity was increased, i.e., the opposite of what was found after 6OHDA lesion.

We did not find strong changes in estimated excitation levels, $G_{E}^{*}$, in the PD rats. Although initially after 6OHDA lesion, loss of dopamine in the striatum increases the excitability of D2 MSNs and decreases the excitability of D1 MSNs, by a month post-lesion homeostatic mechanisms have kicked in to restore the balance (Day et al., 2006; Fieblinger et al., 2014; Shen et al., 2022). D2 MSNs prune cortical and thalamic excitatory synapses to decrease their intrinsic excitability. On the other hand, D1 SPNs do not prune synapses and up-regulate their somatic excitability. These complementary changes restore the excitability balance between the two pathways.

In dyskinetic slices we found enhanced exitability without much change in inhibition, compared to control slices. This is in good agreement with experimental studies. Enhanced glutamatergic input is associated with hyperkinetic disorders (Robelet et al., 2004; André et al., 2010; Sgambato-Faure and Cenci, 2012; Scarduzio et al., 2022). While PD is associated with decreased striatal glutamate, animals treated with L-DOPA to induce dyskinesias displayed a marked increase (Dupre et al., 2011; Nevalainen et al., 2013; Scarduzio et al., 2022). Indeed, the only FDA-approved treatment for LID is amantadine, a drug with NMDA receptor antagonist properties. mGluR5 antagonism and NAMs attenuate LID in both preclinical models and patients (Sebastianutto and Cenci, 2018; Pourmirbabaei et al., 2019). Persistent LTP, loss of LTD and depotentiation at corticostriatal synapses has also been found in LID (Belujon et al., 2010; Fieblinger et al., 2014; Thiele et al., 2014; Calabresi et al., 2016). Furthermore chronic L-DOPA administration causes hyperphosphorylation of striatal NMDA receptors (Oh et al., 1998, 1999; Dunah et al., 2000).

Also very interestingly we found that the control slices, CT and MICE, were situated in a strongly fluctuating regime, close to a transition to WTA like activity. In our previous work (Ponzi and Wickens, 2012, 2013, 2022; Ponzi et al., 2020), we, and others (Angulo-Garcia et al., 2016), have shown that such MSN network dynamics is optimal because it consists of slow large coherent fluctuations which are complex and fairly high dimensional, but also highly reproducible if the network is driven by repeated sequences of stimuli. These coherent network dynamics manifest as sequences of cell assemblies which can be utilized in behavior, for example as liquid central pattern generators (CPGs), and for explore/exploit behavior, useful in reinforcement learning tasks. The proximity to the WTA like regime gives network dynamics the appearance of metastable switching between fixed points (Rabinovich and Varona, 2011). Experiments have shown that such recurrent alternating assemblies correlate with behavior (Carrillo-Reid et al., 2008, 2015) and similar activity has been observed in CPGs (Grillner, 1985, 2006).

Intriguingly we found that the reduced inhibition, $G_{I}^{*}$, estimated from PD slices positioned these slices even closer to the full WTA regime. Thus network dynamics gets stuck close to fixed points for longer periods, which manifests as longer lasting dominant cell assemblies, which suppress the rest of the network. Multiple studies have shown PD to be characterized by such striatal hyperactivity and a highly recurrent dominant assembly which monopolizes the microcircuit, reminiscent of what happens when patients cannot move (Jáidar et al., 2010, 2019; Plata et al., 2013b; Pérez-Ortega et al., 2016; Lara-González et al., 2019). We note that this pathological activity could be normalized not only by increasing the inhibition back to normal levels, but also by increasing the excitation (Figure 6E), while maintaining the reduced level of inhibition. Such manipulation would move the activity back into a regime with interval CV similar to control activity (Figure 6E). Indeed it is known that D1 agonists ‘dissolve' the dominant locked in state, without normalizing the enhancement of spontaneous activity (Jáidar et al., 2010). On the other hand L-DOPA, before prolonged application, restores activity to normal conditions (Lemaire et al., 2012; Plata et al., 2013b). It may be that L-DOPA restores the balance between the direct and indirect pathways, not only by normalizing cortical-striatal balance on the direct and indirect pathways but also by normalizing the collateral inhibition (Taverna et al., 2008).

We found that DYS slices exhibited levels of inhibition somewhat between PD and control (CT, MICE) preparations, but also strongly enhanced excitation. This moved the DYS slices away from the WTA regime. The interval CV was normalized closer to CT compared to the PD slices (Figure 6E), but at the expense of increased activity compared to control CT (Figure 6C). This produced short-lived fractured assembly dynamics with multiple transitions (Figure 3D). Such neural assembly multiplication with increased transitions is shown in L-DOPA induced hyperkinetic conditions (Pérez-Ortega et al., 2016; Calderón et al., 2022) again reminiscent of patients showing enhancement of stereotyped hyperkinetic involuntary movements.

We found control slices were close to a transition from a strongly fluctuating dynamical regime to a fixed point WTA like dynamical regime. This regime may optimize its computational properties (Ponzi and Wickens, 2012, 2013, 2022; Ponzi et al., 2020) as a neural reservoir. The complex dynamical patterns generated by recurrent network ‘reservoirs' have been utilized for various computational purposes (Buonomano and Merzenich, 1995; Jaeger, 2001; Maass et al., 2002; Jaeger and Haas, 2004). Computational properties are often found to be optimal when such reservoirs operate close to the “edge of chaos” (Bertschinger and Natschläager, 2004; Legenstein and Maass, 2007; Sussillo and Abbott, 2009; Ponzi and Wickens, 2012, 2013). There are many indications that strong coherent internally generated brain fluctuations are needed for various cognitive and learning processes and may be generated by determinstic brain network chaos. Resting state activity throughout the brain shows strong coherent spontaneous fluctuations driven by the coordinated activity patterns of many cells (Pachitariu et al., 2015; Stringer et al., 2016). Multiple studies have suggested neural dynamics is “critical” (Beggs and Plenz, 2003; Chialvo, 2004) and resides in a regime close to losing stability. Our work suggests that pathology like PD may move the network out of an optimal regime for computation, and while L-DOPA normalizes activity to some degree by normalizing inhibition, it can also “overshoot” and generate too much excitation.

The successful application of SBI in this study highlights its utility in inferring complex model parameters from experimental data. Leveraging advances in deep learning, SBI overcomes challenges associated with likelihood function estimation, providing a robust framework for parameter inference in biophysically detailed models. Moreover, SBI offers the advantage of estimating full distributions over model parameters, offering insights into parameter interactions and uncertainties.

The efficacy of SBI has been demonstrated in various studies across neuroscience research domains. Noteworthy examples include its application in identifying mechanistic models of neural dynamics (Gonçalves et al., 2020), whole-brain network modeling of epilepsy (Hashemi et al., 2022), computational connectomics (Boelts et al., 2023), spectral graph modeling for brain oscillations (Jin et al., 2023), parameterization of multi-compartmental neuron models (Kaiser et al., 2023), and neural posterior estimation in neural mass models (Rodrigues et al., 2021). These studies attest to the versatility and effectiveness of SBI in addressing diverse research questions and modeling challenges within the field of neuroscience.

The main drawback of the current work is that it does not go far enough. In particular due to computational constraints we were not able to vary further network and cellular parameters. Although we were able to find excellent fits to the control experimental slice conditions from within the current parameter ranges, there are multiple parameters which could be varied in future work, such as the connection probability between MSNs, synaptic timescales and short-term plasticity parameters, as well as MSN cellular ion-channel peak conductances known to be affected by dopamine. These modifications may potentially provide better fits in the pathological conditions. The most important extension for future work is the inclusion of D1 and D2 MSNs with different levels of mutual collateral inhibition (Taverna et al., 2008), and different levels of cortical excitation, and their differential modulation by dopamine.

In summary, our findings contribute to a deeper understanding of MSN network dynamics and highlight the potential of SBI as a powerful tool for parameter inference in complex neural models. By elucidating the intricate interplay between model parameters and experimental observations, this study lays the groundwork for a pipeline for future investigations into striatal function and dysfunction.

## Data availability statement

The raw data supporting the conclusions of this article will be made available by the authors, without undue reservation.

## Ethics statement

The animal study was approved by Institutional Committee for Laboratory Animals Care and Use of the Instituto de Fisiología Celular (IFC), UNAM (NOM-062-Z00-1999; project protocol JBD-59-15). The study was conducted in accordance with the local legislation and institutional requirements.

## Author contributions

AC: Formal analysis, Investigation, Methodology, Software, Validation, Visualization, Writing – original draft, Writing – review & editing. AP: Formal analysis, Investigation, Methodology, Software, Validation, Visualization, Writing – original draft, Writing – review & editing, Conceptualization, Supervision. VC: Data curation, Methodology, Writing – original draft, Writing – review & editing. RM: Funding acquisition, Supervision, Validation, Writing – original draft, Writing – review & editing.
