# Supplementary material for: Pathological cell assembly dynamics in a striatal MSN network model
Source: Front Comput Neurosci. 2024 Jun 6;18:1410335. doi: 10.3389/fncom.2024.1410335 (PMC11188713; doi:10.3389/fncom.2024.1410335)
Supplement: Supplementary file 1 [file Data_Sheet_1.pdf]

Pathological cell assembly dynamics in a striatal MSN network model. Supplemental Materials

Astrid Correa[1‡], Adam Ponzi[1,3‡*], Vladimir M. Calderón[2], Rosanna Migliore[1],

1 Institute of Biophysics, National Research Council, Palermo, Italy
2 National Autonomous University of Mexico, Neurobiology Institute, Department of Developmental Neurobiology and Neurophysiology, Querétaro, Mexico
3 Center for Human Nature, Artificial Intelligence, and Neuroscience, Hokkaido University, Sapporo, Japan.

‡These authors contributed equally to this work.
* apdp@chain.hokudai.ac.jp

Supplemental Figures

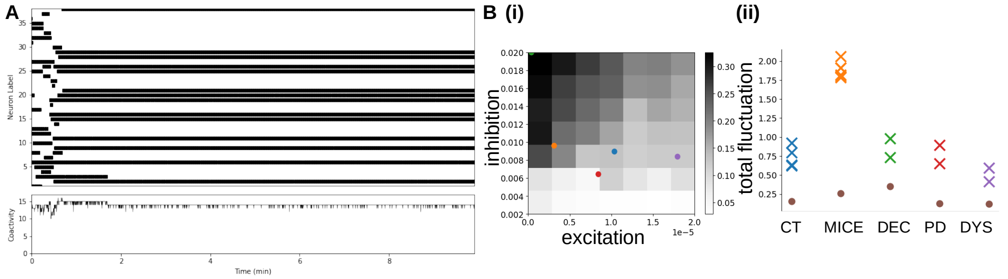

Fig S1. *(a) Example of pathological network simulation in the WTA regime. Parameters are $G_I = 0.0038$, $G_E = 0.85 \times 10^{-5}$. (b) Total fluctuation of the average across cell activity. (i) Dependence of activity features on network simulation parameters inhibition, G_I, and excitation, G_E. Coloured points indicate the average estimated network parameters from slices, blue: CT, orange: MICE, green: DEC, red: PD, purple: DYS. (DEC only half visible in top-left corner). (ii) Coloured crosses: total fluctuation values for individual experimental slice preparations. Brown circles: total fluctuation values calculated from the best fit simulations shown in Figure 3.*

Supplemental Methods

All simulations had 400 cells. Synapses were made randomly between MSNs with probability 0.4, so that each MSN had on average 160 incoming connections with a binomial distribution. For the cell we used the MSN single compartment model developed by Mahon et al. [4], without modification,

$$C\frac{dV}{dt} = -I_{Na} - I_K - I_{Kir} - I_{Af} - I_{As} - I_{Krp} - I_{NaP} - I_{NaS} - I_{syn} - I_{leak} + I_{inj}$$

where V (mV) is the membrane potential, capacitance $C = 1$, ($\mu F cm^{-2}$). In addition to sodium, (I_{Na}) and pottasium, (I_K) channels, the cell model included an inwardly

rectifying potassium current (I_{Kir}), a fast (I_{Af}), and a slowly inactivating A- current (I_{As}) a persistent (I_{Krp}) potassium current, a slowly inactivating (I_{NaS}), and a persistent (I_{NaP}) sodium current, as well as a leak current, $I_{leak} = 0.075(V + 75)$. Ion channel currents obeyed, $I(t) = \bar{g}m^k h(V - E_{cur})$ where E_{cur} (mV) is the ion channel reversal potential, and $\bar{g}$ ($mScm^{-2}$) is the maximal conductance. The gating variables obeyed,

$$\begin{aligned} \frac{dm}{dt} &= (S_m(V) - m)/\tau_m(V) \\ \frac{dh}{dt} &= (S_h(V) - h)/\tau_h(V) \end{aligned}$$

where, $S(V) = 1/(1 + e^{-(V-V_S)/k_s})$ and $\tau(V) = \tau_0/(e^{-(V-V_\tau)/k_\tau} + e^{(V-V_\tau)/k_\tau})$. The parameters $\bar{g}$, E_{cur}, k, V_S, (mV) k_s, (mV) V_τ, (mV) k_τ (mV) and τ_0 (ms), for each of the ion-channels were as described in [4], Table 1 and the text.

The cell excitability was modeled using somatic current injection, I_{inj}, ($mAcm^{-2}$) using Neuron IClamp. For any given simulation, a random variable G_E, was uniformly drawn from the range $[0, 0.00002]$ and all MSN cells in the simulation were driven above threshold by somatic current injection, I_{inj}, with uniform random strength between rheobase 0.001305 and $0.001305 + G_E$ which was fixed for the duration of a simulation.

Synaptic currents, I_{syn}, between MSNs were given by a dual exponential [1–3] model,

$$I_{syn}(t) = g(t)(V(t) - E_{rev})$$

where $g(t) = (g_1(t) - g_2(t))$ is the synaptic conductance, composed of rising, $g_2(t)$ and decaying $g_1(t)$ components, $V(t)$ is the postsynaptic membrane potential and $E_{rev} = -85$ mV is the synaptic reversal potential. Synaptic conductances evolve according to,

$$\begin{aligned} \frac{dg_1}{dt} &= -g_1(t)/\tau_{decay} + AGS^+(t)R^-(t)\delta(t - t_{sp}) \\ \frac{dg_2}{dt} &= -g_2(t)/\tau_{rise} + AGS^+(t)R^-(t)\delta(t - t_{sp}) \end{aligned}$$

Here t_{sp} is the presynaptic spike arrival time (after a delay - see below), G is the synaptic weight (see below), $\tau_{rise} = 0.2$ ms and $\tau_{decay} = 20$ ms are the IPSC rise and decay time constants, respectively, and $A = e^{-t_p/\tau_{decay}} - e^{-t_p/\tau_{rise}}$ is a normalization constant so that peak conductance is attained at $t_p = (\tau_{decay}\tau_{rise})/((\tau_{decay} - \tau_{rise})\log(\tau_{decay}/\tau_{rise}))$. $S(t)$ and $R(t)$ are the short-term plasticity variables, and $S^\pm, R^\pm$, denote their values just before (-) and after (+) the spike. The Tsodyks-Markram model [1–3] assumes that each synapse has a pool of available neurotransmitter resources $R(t)$ that is utilized by a presynaptic action potential with a release probability $S(t)$. $R(t)$ decreases and $S(t)$ increases after an action potential and they both recover between spikes to steady-state values. The speed of recovery of the short-term plasticity variables is given by time constants D and F (ms) that together determine the short-term dynamics of the synapse,

$$\begin{aligned} \frac{dR}{dt} &= \frac{(1 - R(t))}{D} - S^+(t)R^-(t)\delta(t - t_{sp}) \\ \frac{dS}{dt} &= -\frac{S(t)}{F} + U(1 - S^-(t))\delta(t - t_{sp}) \end{aligned}$$

where U is the utilization of synaptic efficacy or absolute release probability. Here we set the synaptic utilization factor (U), 0.41, depression time constant (D), 222 ms, and facilitation time constant (F), 1859 ms. For any given simulation, the strength of lateral

inhibition, G_I, was uniformly drawn from the range $[0.002, 0.02]$. All MSN synaptic weights, G, were uniformly random in the range $0.3125G_I$ to $0.9375G_I$, $(mScm^{-2})$. MSN-MSN synapses also included fixed delays which varied uniform randomly between 1 and 3 msec.

References

1. Ecker, A., Romani, A., Sáray, S., Káli, S., Migliore, M., Falck, J., Lange, S., Mercer, A., Thomson, A.M., Muller, E. and Reimann, M.W., 2020. Data-driven integration of hippocampal CA1 synaptic physiology in silico. Hippocampus, 30(11), pp.1129-1145.

2. Fuhrmann, G., Segev, I., Markram, H. and Tsodyks, M., 2002. Coding of temporal information by activity-dependent synapses. Journal of neurophysiology, 87(1), pp.140-148.

3. Tsodyks MV and Markram H, 1997. The neural code between neocortical pyramidal neurons depends on neurotransmitter release probability. Proceedings of the national academy of sciences, 94(2), pp.719-723.

4. Mahon S, Deniau JM, Charpier S, Delord B. Role of a striatal slowly inactivating potassium current in short-term facilitation of corticostriatal inputs: a computer simulation study. Learning & Memory. 2000 Sep 1;7(5):357-62.
